# Developing cell-based therapies for pancreatic ductal adenocarcinoma

**DOI:** 10.1172/JCI189513

**Published:** 2025-04-15

**Authors:** Rachel Elizabeth Ann Fincham, Joe Poh Sheng Yeong, Hemant Mahendrakumar Kocher

**Affiliations:** 1 Centre for Tumour Biology, Barts Cancer Institute, Queen Mary University of London, London, United Kingdom.; 2 Institute of Molecular and Cell Biology (IMCB), Agency for Science, Technology and Research (A*STAR), Singapore.; 3 Centre for Quantitative Medicine, Duke-National University of Singapore (NUS) Medical School, Singapore.; 4 Cancer Science Institute of Singapore, National University of Singapore, Singapore.; 5 Barts and the London Hepato-Pancreato-Biliary (HPB) Centre, The Royal London Hospital, Barts Health National Health Service Trust, London, United Kingdom.

## Abstract

Prostate stem cell antigen (PSCA) is highly and preferentially expressed on the surface of pancreatic ductal adenocarcinoma (PDAC) cells, raising the promise of tumor-selective cell-based immunotherapies. In this issue of the *JCI*, Dai et al. harness PSCA for the development of an off-the-shelf chimeric antigen receptor (CAR) invariant natural killer T (iNKT) cell–based treatment for PDAC. Through in vitro experiments and in vivo models, the authors demonstrate selectivity and therapeutic efficacy of PSCA CAR_sIL15 iNKT cells against both gemcitabine-sensitive and -resistant PDAC cells with comparable antitumor activity for freshly produced and frozen off-the-shelf PSCA CAR_sIL15 iNKT cells. This development opens another potential therapeutic option for pancreatic cancer.

## Pancreatic ductal adenocarcinoma treatment

Pancreatic ductal adenocarcinoma (PDAC) remains one of medicine’s largest areas of unmet need. Surgery is the only curative option for patients with PDAC; however, with late-stage disease presentation, only 15%–20% of patients are eligible for surgical removal of localized cancer. While targeted therapies have improved patient prognosis in other cancers, their efficacy is limited for patients with PDAC (1). As such, standard chemotherapy continues to be the cornerstone of treatment, remaining unchanged for nearly a decade (1). Thus, alternative therapeutic methods for PDAC are desperately needed. Stromal targeting and cell-based immunotherapies are attractive due to the characteristics of PDAC (2–4).

## Stromal targeting and immune based therapies in PDAC

Characterized by its dense desmoplasia, the PDAC tumor microenvironment (TME) is highly immunosuppressive. Largely orchestrated by stromal pancreatic stellate cells (PSCs), the desmoplastic reaction observed in PDAC results in tumor hypoxia, a key factor in disease aggressiveness and chemotherapy resistance (5). Moreover, sequestration of immune cells, including CD8^+^ T cells, B cells, and natural killer (NK) cells, limits effective immune surveillance (6–8). Consequently, recent trials have investigated stromal targeting agents as well as immune cell–based therapies for the treatment of PDAC. For example, in the phase Ib Stromal TARgeting for PAncreatic Cancer (STARPAC) trial, all-trans retinoic acid (ATRA) was used as a stromal targeting agent to render PSCs quiescent, rather than eliminate them, as well as modulate immune response as measured by pentraxin-3 (9, 10). ATRA in combination with gemcitabine-Nab-paclitaxel was found to be safe and tolerable in patients with advanced, unresectable PDAC, leading to a phase II trial, STARPAC2 (11). Clinical development of preclinically promising stromal-targeting and immune-modifying agents, such as pegvorhyaluronidase Aalfa, when combined with standard chemotherapy regimen (Nab-paclitaxel plus gemcitabine) has not lived up to its hope (12). Moreover, the spatial distribution of immune cells, such as NK cells, can be altered in response to stromal targeting in murine models of PDAC (6). Taken together, these results suggest that modulation of the TME and the subsequent cell-cell interactions in PDAC may have prognostic implications.

Cellular therapies, such as chimeric antigen receptor (CAR) T cells have demonstrated striking efficacy in hematological malignancies and thus have received much attention as a potential treatment for solid tumors, including PDAC, with targets such as CEACAM7 and Claudin18.2 (3, 13). However, substantial challenges persist, including the effective trafficking of CAR-T cells to sites of malignancy. Furthermore, the heterogeneous PDAC TME may result in cellular dysfunction and exhaustion upon arrival (14, 15). Notably, one of the largest challenges facing CAR-T cell therapy is graft-versus-host disease (GvHD) in which the transplanted cells react to off-target, normal patient tissue, leading to adverse events (16).

Since the development of autologous CAR-T cells is expensive and time consuming and further work is required to reduce potential GvHD invoked by allogenic CAR-T cells, attention has fallen on alternative cell types as the foundation for cell-based therapies. Thus, NK and natural killer T (NKT) cells with a unique opportunity to reduce GvHD, while exhibiting rapid inherent cytolytic capabilities, have been developed to enhance the safety and efficacy of CAR-targeted cell therapy (17, 18).

**Figure 1:**
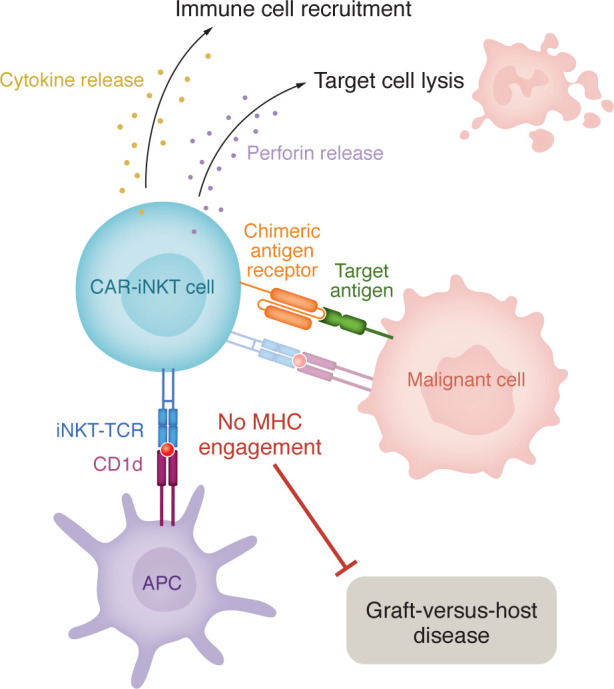
CAR-invariant NKT cells engage via the CD1d receptor on malignant and antigen-presenting cells. CAR-invariant NKT cells target malignant cells through engagement of their CAR with the target antigen present on the cancer cell surface. Binding induces rapid cytokine release, stimulating recruitment of additional immune cell subsets, such as T cells and NK cells. In addition, CAR-iNKT cells induce direct tumor cell cytolysis through perforin release. CAR-iNKT cells may also engage with malignant cells through invariant NKT TCR engagement with tumor antigens presented via the CD1d receptor. While some tumoral cells express CD1d, this receptor-ligand interaction is largely mediated by antigen-presenting cells. Signaling through CD1d does not involve engagement of the MHCs and thus prevents induction of GvHD, a common adverse event observed in response to allogeneic CAR-T cell treatment.

The distinct immune cell subset NKT cells are characterized by an invariant α-chain T cell receptor. Unlike T cells, NKT cells engage in target recognition through engagement of the CD1d receptor on antigen presenting cells (Figure 1) (19). This mechanism of action limits the risk of GvHD typically induced by major histocompatibility complex (MHC) class I involvement. NKT cells bridge the gap between the innate and adaptive immune systems, possessing several NK-like functions, such as rapid cytokine secretion and cellular cytotoxicity (17, 18). Additionally, these effector cells are capable of intricate cellular crosstalk between cells of both the innate and adaptive immune system, promoting activation of dendritic and NK cells, B and T cells, as well as macrophages and neutrophils (20). Taken together, these attributes make NKT cells a very attractive therapeutic option.

## PSCA CAR_sIL-15 iNKT cells in PDAC

Recent evidence has revealed prostate stem cell antigen (PSCA) as a key diagnostic biomarker in PDAC, with approximately 30%–60% of PDAC exhibiting overexpression, with almost no expression in normal tissues (21, 22). Thus, this biomarker may also be an effective target for cell-based therapy (23, 24).

In this issue of the *JCI*, Dai et al. leveraged PSCA to produce invariant CAR NKT cells derived from peripheral blood mononuclear cells (25). Combined with a soluble IL-15 (sIL-15) element, which has previously been shown to prevent invariant NKT (iNKT) cell inhibition by macrophage cells and/or hypoxia, Dai et al. developed an off-the-shelf iNKT product that would enhance the natural antitumor effects of iNKT cells without inducing toxicity. Their elegant design included a truncated epidermal growth factor receptor (EGFR) component, which would act as a safety switch to deplete the PSCA CAR_sIL15 iNKT cells upon administration of cetuximab, a monoclonal antibody targeting EGFR. In vitro assays revealed that PSCA CAR_sIL15 iNKT cells expressed higher levels of the activation markers CD69 and CD25 when compared with CAR_sIL15 iNKT cells. Moreover, this effect was shown to be PSCA dependent, with no upregulation of CD69/CD25 observed in response to the PSCA^–^ cell line BxPC3. Similar trends were noted in PSCA CAR_sIL15 iNKT degranulation, cytolytic function, and cytokine release. Metastatic and orthotopic models further revealed antitumor activity of PSCA CAR_sIL15 iNKT cells, with superior efficacy observed in orthotopic models. Notably, Dai et al. also observed the upregulation of PSCA in gemcitabine-resistant cells and further demonstrated PSCA CAR_sIL15 iNKT cell efficacy in gemcitabine-resistant PDAC models (25).

Importantly, this work demonstrates consistent efficacy between freshly produced and frozen off-the-shelf PSCA CAR_sIL15 iNKT cells (25). Thus, using optimized expansion platforms, large-scale production of this cellular therapy may be possible for use in clinical trials and ultimately patient care. Moreover, the authors report similar efficacy between off-the-shelf CAR_sIL15 iNKT cells and conventional PSCA CAR_sIL15 T cells. Importantly however, unlike mice treated with PSCA CAR_sIL15 T cells, those treated with PSCA CAR_sIL15 iNKT cells did not demonstrate resultant GvHD or cytokine release syndrome. Taken together, the authors present compelling findings that suggest a role for PSCA CAR_sIL15 iNKT cells in the treatment of pancreatic cancer, a therapy which may be particularly relevant for gemcitabine-resistant patients (25).

## Discussion and future directions

While this study presents an elegant design and exciting findings, several factors must be considered (25). Both the metastatic and orthotopic murine models displayed within the study are reliant on mice from a Nod-Scid-Gamma (NSG) background, which are not representative of the complex cell-cell interactions observed within PDAC TME. This paradigm was further reinforced using cell-derived xenograft (CDX) models, which also relied on NSG mice. However, these models lack the cellular and stromal heterogeneity observed within patient-derived models (13). Indeed, within the field of pancreatic cancer, further development of appropriate murine models is necessary to successfully recapitulate and investigate the complex TME interactions observed within this disease, especially since PDAC TME may alter, if not hinder, trafficking of immune cells. Furthermore, although PSCA has been suggested as a biomarker for PDAC, the authors note that PDAC cell lines exhibit variable expression of PSCA, with Capan-1, MIA-PaCa2, and Aspc-1 cells demonstrating high PSCA expression, while Panc-1 and BxPC3 were classified as PSCA^–^. With only 30%–60% of patients expressing PSCA, the clinical application of this therapy may be limited. Nevertheless, Dai et al. (25) provide crucial insight into an exciting therapeutic for the treatment of PDAC. With striking response rates observed in all models tested, and with 100% survival at day 80 being reported, the remarkable efficacy of PSCA CAR_sIL15 iNKT cells is compelling. Dai et al. (25) provide a firm foundation from which to further explore and validate this cellular therapy in additional preclinical models. If these results are replicated, an exciting clinical trial will be on the horizon.

## Version 1 04/15/2025

Electronic publication
